# Prevalence and Sociodemographic Correlates of Mental Health Symptoms Among Canadian Youth: A Cross-Sectional Study

**DOI:** 10.1177/13591045261446034

**Published:** 2026-04-22

**Authors:** Ogochukwu Kelechi Onyeso, Adebiyi G. Boco, Md Kamrul Islam, Lars K. Hallstrom

**Affiliations:** 1 Prentice Institute for Global Population and Economy, 4512 University of Lethbridge, Lethbridge, AB, Canada; 2 Health Analysis and Modelling Division, Statistics Canada, Ottawa, ON, Canada

**Keywords:** adolescent, anxiety disorders, depression and mood disorders, psychological stress, mental health

## Abstract

**Background:**

Recent data highlight problematic rates of mental health symptoms (MHS) among adolescents and young adults following the COVID-19 pandemic, with implications for early identification and clinical service provision. This study estimated the prevalence of MHS and its sociodemographic correlates among Canadian youths.

**Methods:**

We conducted a cross-sectional analysis of 896 iundividuals aged 18–24 years using data from Statistics Canada’s Survey on COVID-19 and Mental Health. Symptoms of anxiety, psychological distress, depression, and post-traumatic stress disorder (PTSD) were assessed using validated self-report instruments. Analyses included descriptive statistics, Pearson’s chi-square tests, two-way analysis of covariance (ANCOVA), and multivariate linear regression.

**Results:**

Participants’ mean (SD) age was 21.2 (2.0) years; 53% identified as women, and 81% lived in urban areas. Prevalence was highest for psychological distress (57.0%), followed by depression (31.6%), anxiety (23.7%), and PTSD symptoms (12.6%). Women reported higher anxiety and distress than men (*p* < 0.05). In multivariable analyses, female gender, non-essential worker status, poorer self-rated mental health, weaker community belonging, lower household income, lower life satisfaction, and pre-existing mental health disorder were associated with greater symptom severity (*p* < 0.05).

**Conclusion:**

The rising prevalence of mental health symptoms reflects gendered, socioeconomic, and psychosocial vulnerabilities, requiring trauma-informed clinical screening and equity-focused public health interventions for Canadian youth.

## Plain Language Summary

Before the COVID-19 pandemic, approximately one in five Canadian youth experienced mental health symptoms annually, with limited access to appropriate care; only 20% received the help they needed. Hospitalization rates for mental health conditions were also increasing, signaling a growing public health concern. This cross-sectional study examines the prevalence and sociodemographic correlates of mental health symptoms among Canadian youth aged 18 to 24 during the COVID-19 pandemic. Using national survey data, we found that about 60% of respondents met the screening criteria for at least one mental health disorder, indicating a significant rise compared with pre-pandemic levels. Psychological distress was the most common condition, followed by symptoms of anxiety and depression. The findings revealed that women and youth experiencing socioeconomic or psychosocial disadvantages were disproportionately affected, reflecting intersecting vulnerabilities that compound mental health risks. In contrast, essential workers reported lower psychological distress levels, possibly due to heightened health awareness, stronger coping mechanisms, and a sense of social purpose linked to their occupational roles. These results underscore the urgent need for targeted mental health interventions, particularly for socioeconomically disadvantaged groups and young women. Strengthening accessibility, affordability, and inclusiveness of mental health services could mitigate long-term adverse outcomes. Moreover, identifying the youth demography at most risk may guide the development of community-based preventive and support strategies to enhance youth mental well-being during future public health crises.

## Introduction

For over three decades, mental health has remained a pressing concern among Canadian children and youth. National statistics estimate that nearly one in five young people meet the criteria for at least one mental health disorder each year (Statistics Canada, 2021a; Vaillancourt et al., 2021). The COVID-19 pandemic, by disrupting education, employment, and social connections, has intensified these challenges (Bulloch et al., 2021; Capaldi et al., 2022). Emerging reports document sharp increases in depression, anxiety, psychological distress, and post-traumatic stress disorder (PTSD) among young adults during this period (Craig et al., 2022; Gill et al., 2022; Hawke et al., 2020).

Although the literature consistently signals rising rates of psychological symptoms, the majority of Canadian studies have methodological limitations. Several relied on a small sample (Gill et al., 2022), convenience sampling (Craig et al., 2022), was confined to a single university setting (King et al., 2022), or were restricted to particular provinces (Hawke et al., 2020; Salmon et al., 2022). Even when larger samples were included, analyses rarely extended to the sociodemographic correlates of mental health or the ways in which these factors intersect (Gill et al., 2022). This leaves a limited understanding of how gender, income, race, Indigeneity, and other social factors interact to shape youth mental health during crisis periods. A nationwide estimate that integrates these dimensions is therefore essential.

Youths represent a particularly vulnerable group during health crises (McGorry et al., 2025); they face major transitions, completing education, entering the workforce, and establishing independence, while often lacking stable financial and social resources (Ludwig-Walz et al., 2023; Vaillancourt et al., 2021). During the pandemic, these vulnerabilities were exacerbated by school closures, job losses, and disruptions to peer and community networks (Hawke et al., 2020). Our previous study showed a significant effect of the pandemic’s social and economic disruptions on the youth mental health symptoms (Onyeso et al., 2025). The consequences are wide-ranging, including heightened risks of substance use, sleep disturbances, cognitive impairment, family conflict, and suicidal ideation (Bourmistrova et al., 2022; Guerrero & Barnes, 2022; Premraj et al., 2022). Importantly, stigma around mental illness continues to deter youth from seeking help, contributing to unmet need and long-term negative outcomes (Xiong et al., 2020).

Mental health challenges are not evenly distributed. Pre-pandemic research shows that women, youth from low-income households, and racialized or immigrant populations report higher rates of distress (Findlay et al., 2020; Mental Health Commission of Canada, 2021). Yet little is known about how these vulnerabilities compounded during the pandemic, or whether structural inequalities such as gender and place of residence interact to magnify risk (Capaldi et al., 2022; Salmon et al., 2022). International reviews emphasize that young adults should be prioritized in recovery planning and explicitly considered in strategies for future public health emergencies (Newlove-Delgado et al., 2023). To do so, research must move beyond documenting prevalence to examining how sociodemographic factors shape outcomes and create overlapping vulnerabilities.

This study is grounded in the Social Determinants of Health (SDH) framework (World Health Organization, 2022), which emphasizes that health outcomes are shaped not only by individual behaviours but by broader social, economic, and environmental conditions. For youth, determinants such as income security, education, employment, housing, and social belonging are particularly salient. The pandemic highlighted how disruptions in these domains, such as reduced work hours, remote learning challenges, and weakened community ties, exposed young adults to increased mental health risks (Onyeso et al., 2025; Yu et al., 2021). Applying the SDH framework allows us to interpret prevalence not merely as an epidemiological measure, but as the outcome of structural inequities. This lens underscores the need to identify which youth populations are most disadvantaged and to inform policies that reduce barriers to mental health and social supports.

Building on these gaps and guided by the SDH framework, the present study aims to: (1) estimate the prevalence of four mental health symptoms; anxiety, depression, psychological distress, and PTSD among Canadian young adults aged 18 to 24 during the COVID-19 pandemic, (2) examine the influence of sociodemographic characteristics, including gender, education, area of residence, indigeneity, visible minority status, immigration status, household income, community belonging, and self-rated health, and (3) explore whether intersectional effects, particularly between gender and area of residence, further explain variations in mental health symptoms.

By offering a nationwide analysis of mental health symptom prevalence and sociodemographic correlates, this study extends the Canadian evidence base in several ways. First, it overcomes limitations of earlier pandemic research by drawing on Statistics Canada’s Survey on COVID-19 and Mental Health, a large-scale dataset with robust methodology. Second, it provides an intersectional analysis, highlighting how overlapping vulnerabilities shape risk. Finally, it translates findings into actionable insights for policy, particularly the importance of gender-stratified and equity-focused approaches in post-pandemic public health surveillance. Hence, the study contributes to both scholarly debates and practical efforts to support the well-being of Canadian youth.

## Methods

### Data Source

The study was a secondary data analysis using Statistics Canada’s Survey on COVID-19 and Mental Health (SCMH) datasets (2020 and 2021) (Statistics Canada, 2023). The SCMH was a cross-sectional, national rapid-response survey to assess the impacts of COVID-19 on Canadians’ mental health and well-being (Statistics Canada, 2023). The SCMH employed a simple random two-stage sampling design: the sampling unit for the first stage was the dwelling within each province, and the second stage was the persons within a dwelling (Statistics Canada, 2023). Statistics Canada obtained informed consent from the participants and was responsible for the research procedures and ethics.

The present study pooled the two cycles of SCMH collected in the fall of 2020 (*n* = 14,689) and spring of 2021 (*n* = 8,032) (Guerrero & Barnes, 2022). The survey was administered online or by telephone. Aside from marital status, which was not collected in 2020, the questions, variables, mode of administration, and the study population were the same for both cycles. Therefore, the pooled dataset met all five assumptions of comparability: same study population, sampling technique, instrument, outcomes, and context (Schenker & Raghunathan, 2007), and Thomas and Wannell (2009) guidelines for pooling similar Statistics Canada datasets. SCMH involved individuals aged 18 years and older from all provinces and territories in Canada, excluding people living on federal reserves, in institutions and outside the capital cities in the territories. The study involved young adults aged 18 to 24 years. After rigorous data cleaning, including removing univariate and multivariate outliers based on the primary outcomes, the study involved 896 participants.

### Patient and Public Involvement

Our study involved a secondary analysis of the Statistics Canada Mental Health (SCMH) survey data for 2020 and 2021. Statistics Canada promotes public involvement in their survey designs by establishing multi-stakeholder steering committees during the development phase of their surveys. For this research, the study proposal was shared internally with academic colleagues for feedback during the development and review stages. Interpretation of findings was informed by scholarly discussion and peer feedback, including presentations at relevant academic conferences. This engagement process allowed us to ensure that our research was in tune with community interests and that the results were communicated in an effective manner.

### Outcome Variables

The SCMH used standardized screening tools to assess symptoms associated with anxiety, psychological distress, depression, and post-traumatic stress disorder (PTSD) (Statistics Canada, 2021b). Anxiety was assessed using the Generalized Anxiety Disorder Scale (GAD-7) (Spitzer et al., 2006). The GAD-7 is a seven-item questionnaire that evaluates the presence and severity of generalized anxiety disorder. Items were rated on a four-point Likert scale ranging from 0 (not at all) to 3 (nearly every day). The responses were summed to provide a score ranging from 0 to 21, with higher scores indicating greater anxiety levels. A score of 10 or higher represented the cut-off point for identifying cases of generalized anxiety disorder (Guerrero & Barnes, 2022; Spitzer et al., 2006).

Psychological distress was assessed using the ten-item Kessler Psychological Distress Scale (K10) (Kessler et al., 2003). The scale obtained respondents’ self-rating of their psychological distress symptoms in the previous four weeks based on a five-point Likert scale ranging from 0 (none of the time) to 4 (all of the time). The expected range was 0 to 40, with higher scores indicating greater levels of psychological distress (Cornelius et al., 2013). Nine (9) was adopted as a cut-off mark previously used in the Canadian context (Caron & Liu, 2010; Guerrero & Barnes, 2022).

Depressive symptoms were assessed using the nine-item Patient Health Questionnaire (PHQ-9) (Kroenke et al., 2001; Manea et al., 2012). The PHQ-9 assesses the frequency with which nine depressive symptoms have occurred in the past two weeks on a four-point Likert scale ranging from 0 (not at all) to 3 (nearly every day). Respondents’ overall scores range from 0 to 27, with higher scores indicating more severe depressive symptoms. A score of 10 was the cut-off point for identifying cases of major depressive disorder (Kroenke et al., 2001; Manea et al., 2012). The PTSD was assessed using the 20-item self-report PTSD checklist (PCL-5), a derivative of the Diagnostic and Statistical Manual of Mental Disorders Version 5 (Roberts et al., 2021). The PCL-5 is a five-point Likert scale (0 = not at all, 4 = extremely) describing an individual’s traumatic experiences. The total score can range from 0 to 80. Thirty-three (33) was adopted as the cut-off point following the recommendations in the literature (Bovin et al., 2016).

### Covariates

Following an in-depth literature review, 16 covariates were included in the models: age, gender, level of education, area of residence, immigration status, indigenous identity, visible minority identity, COVID-19 locus, sense of belonging to the local community, history of access to mental and emotional health resources, perceived mental health, mental health status compared to before COVID-19, being an essential health worker, household size, total household income (THI), satisfaction with life in general, and region of residence. An essential worker is an individual who works in a service, facility or activity necessary for the preservation of life, health, public safety, or basic societal functions (Guerrero & Barnes, 2022). Satisfaction with life in general was self-reported on a scale from 1 to 10, with higher scores indicating greater satisfaction.

The COVID-19 locus was a derived variable measuring a participant’s level of social connectedness with people diagnosed with COVID-19 infection. COVID-19 locus was a dichotomous variable defined as “proximal” if the participant or any household member was diagnosed with COVID-19 or “distal” if the participant’s close friend or family member outside the household, a coworker or colleague, or someone else interacted with in the community was diagnosed with COVID-19. This variable aligns with the socioecological framework for mental health (Michaels et al., 2022), allowing us to explore how proximal and distal pandemic-related exposures may differentially influence youth mental health outcomes.

### Statistical Analysis

Data was analyzed using the Statistical Package for Social Sciences (SPSS) Version 28. Descriptive statistics – frequency (percentage) and mean (standard deviation) were used to summarize the participants’ characteristics. The primary outcome variables were dichotomized to facilitate prevalence and crosstabulation analysis. However, continuous outcome variables were used for multivariate regression analysis to avoid the loss of statistical power and inflation of residual confounding inherent in dichotomizing a continuous variable for multivariate analyses (Royston et al., 2006).

The dataset was diagnosed and fixed for univariate and multivariate outliers using the standardized Z-score and Mahalanobis distance approaches. Each mental health outcome was assessed for normality by computing its skewness, linearity was established through a QQ plot, and multicollinearity in each regression model was assessed and adjusted using the variance inflation factor (VIF <5).

Pearson’s Chi-square was used to test the association between mental health indicators and sociodemographic characteristics. A two-way analysis of covariance (ANCOVA) was used to estimate the main and interaction effects of gender and area of residence on the mental health indicators while adjusting for the covariates. Finally, multivariate linear regression models were used to determine the sociodemographic factors that could predict the mental health indicators.

## Results

### Sociodemographic Characteristics

The sample included 896 Canadian young adults aged 18 to 24 years; mean (SD) = 21.15 (2.01). Table 1 shows the samples’ sociodemographic characteristics. Females were 52.9% of the sample. One-third (33.0%) of the participants had a tertiary education, and the majority lived in the urban area (81.3%). Among the respondents, 6.6% identified themselves as Indigenous, 19.5% as immigrants, and 28.2% as visible minorities. Almost half of the sample (44.5%) believed the COVID-19 pandemic worsened their mental health. The average household size and number of people aged 18 years and above per household were four and three, respectively. The average (SD) annual household income was 93903.28 (75201.30) Canadian dollars, and satisfaction with life in general was 6.45 (2.15).

**Table 1.** Sample Sociodemographic Characteristics

Parameter	Frequency (*n* = 896)	Percentage %
Gender		
Male	422	47.1
Female	474	52.9
Highest education level		
High school/equivalent or lower	598	66.7
Tertiary (college, university, postgraduate)	296	33.0
Missing	2	0.3
Area of residence		
Urban	728	81.3
Rural	163	18.2
Missing	5	0.5
Region of residence		
Atlantic	208	23.2
Central	290	32.4
Prairie	229	25.6
West coast	85	9.5
North	84	9.3
Indigenous identity flag		
Indigenous identity	59	6.6
Non-indigenous identity	835	93.2
Missing	2	0.2
Immigration status		
Non-immigrant	716	79.9
Immigrant/Non-permanent resident	175	19.5
Missing	5	0.6
Visibility minority flag		
Visible minority	253	28.2
Not a visible minority	629	70.2
Missing	14	1.6
Perceived mental health		
Poor	62	6.9
Fair	200	22.3
Good	278	31.0
Very good	214	23.9
Excellent	141	15.8
Missing	1	0.1
Perceived mental health compared to before COVID-19		
Worse	399	44.5
Same	415	46.3
Better	82	9.2
COVID-19 locus		
Distal (did not know any diagnosed person)	676	75.4
Proximal (associated with a diagnosed person)	220	24.6
Employed/working during the pandemic		
No/Valid Skip	375	41.9
Yes	517	57.7
Missing	4	0.4
Essential worker		
No/Valid skip/Don’t know	633	70.6
Yes	260	29.0
Missing	3	0.4
Sense of belonging to the local community		
Very strong	81	9.0
Somewhat strong	380	42.4
Somewhat weak	300	33.5
Very weak	130	14.5
Missing	5	0.6
Accessed emotional and mental health resources		
No	664	74.1
Yes	226	25.2
Missing	6	0.7

Source: Survey on COVID-19 and Mental Health (2020/2021).

### Prevalence of Mental Health Symptoms

Table 2 showed that the mental health symptom with the highest prevalence rate was psychological distress (57.0%), followed by depression (31.6%), anxiety (23.7%), and PTSD (12.6%). Over one-half of the sample (59.6%) met at least one of the four mental health symptoms, of which 24.2% met one, 13.6% met two, 13.6% met three, and 8.1% met all four indicators of poor mental health.

**Table 2.** Mental Health Profile of the Participants (Based on Standardized Cut-Off)

Parameter	% Met	% Un-met	% Missing	Total (Freq., %)
Anxiety	23.7%	74.4%	1.9%	896 (100.0%)
Distress	57.0%	43.0%	-	896 (100.0%)
Depression	31.6%	66.3%	2.1%	896 (100.0%)
PTSD	12.6%	84.9%	2.5%	896 (100.0%)

Source: Survey on COVID-19 and Mental Health (2020/2021).

PTSD = post-traumatic stress disorder. Anxiety scale = 0–21, cutoff = 10; Distress scale = 0–40, cutoff [Canadian normative value] = 9; Depression scale = 0–27, cutoff = 10; PTSD scale = 0–80, cutoff = 33. Number of Mental Health Disorders Met: none = 362(40.4%), one = 217(24.2%), two = 122(13.6%), three = 122(13.6%), and met all = 73(8.1%). Percentages were estimated without missing cases; bivariate estimates are based on available case analysis.

### Association Between Demographic Variables and Mental Health Symptoms

The bivariate analysis**,** Pearson’s Chi-square statistics (Table 3), showed that there were significant gender differences in participants who met the cut-off for anxiety (χ^2^ = 27.401, *p* < 0.001), distress (χ^2^ = 34.862, *p* < 0.001), depression (χ^2^ = 29.088, *p* < 0.001), and PTSD (χ^2^ = 27.607, *p* < 0.001), female respondents had worse mental health symptoms than males. There was a significantly higher proportion of distressed (χ^2^ = 16.654, *p* < 0.001) and depressed (χ^2^ = 5.835, *p* = 0.016) people in the urban areas compared with rural areas. Non-immigrants had higher anxiety (χ^2^ = 4.161, *p* = 0.041) and depression (χ^2^ = 7.197, *p* = 0.007) than immigrants and non-permanent residents. There was a significant association (*p* < 0.05) between mental health symptoms and being non-minority, unemployed, and non-essential workers (Table 3).

**Table 3.** Bivariate Association Between Sociodemographic Variables and Mental Health Outcomes

Category	Anxiety Met		Distress Met		Depression Met		PTSD Met	
	*f*	(%)	*f*	(%)	*f*	(%)	*f*	(%)
Gender								
Male	66	16.1	197	46.7	96	23.2	27	6.6
Female	146	31.2	314	66.2	187	40.3	86	18.5
Total	212	24.1	511	57.0	283	32.3	113	12.9
ꭓ^2^ -statistic (df)	27.401 (1)		34.862 (1)		29.088 (1)		27.607 (1)	
*P*-Value	<0.001*		<0.001*		<0.001*		<0.001*	
Education level								
High school/equivalent or lower	142	24.2	338	56.5	190	32.2	70	12.0
Tertiary (college, university, postgraduate)	70	24.0	171	57.8	93	32.5	43	14.8
Total	212	24.1	509	56.9	283	32.3	113	12.9
ꭓ^2^ -statistic (df)	0.007 (1)		0.126 (1)		0.009 (1)		1.390 (1)	
*P*-Value	0.933		0.723		0.926		0.238	
Area of residence								
Urban	180	25.2	440	60.4	244	34.3	87	12.3
Rural	31	19.4	70	42.9	39	24.4	26	16.4
Total	211	24.1	510	57.2	283	32.5	113	13.0
ꭓ^2^ -statistic (df)	2.430 (1)		16.654 (1)		5.835 (1)		1.929 (1)	
*P*-Value	0.119		<0.001*		0.016*		0.165	
Indigenous identity^a^								
Indigenous identity	20	33.9	40	67.8	24	42.1	—	—
Non-indigenous identity	192	23.4	469	56.2	259	31.6	—	—
Total	212	24.1	509	56.9	283	32.3	—	—
ꭓ^2^ -statistic (df)	3.285 (1)		3.039 (1)		2.677 (1)		—	
*P*-Value	0.070		0.081		0.102		—	
Immigration status								
Non-immigrant	180	25.6	420	58.7	242	34.5	92	13.2
Immigrant/Non-permanent resident	31	18.1	89	50.9	41	23.8	21	12.1
Total	211	24.1	509	57.1	283	32.4	113	13.0
ꭓ^2^ -statistic (df)	4.161 (1)		3.495 (1)		7.197 (1)		0.129 (1)	
*P*-Value	0.041*		0.062		0.007*		0.720	
Visibility minority identity								
Visible minority	47	19.0	136	53.8	75	30.2	34	13.5
Not a visible minority	160	25.8	364	57.9	203	33.0	74	12.1
Total	207	23.9	500	56.7	278	32.2	108	12.5
ꭓ^2^ -statistic (df)	4.514 (1)		1.244 (1)		0.596 (1)		0.334 (1)	
*P*-Value	0.034*		0.265		0.440		0.563	
Perceived mental health								
Poor/Fair	139	54.9	242	92.4	176	68.5	77	30.7
Good/Very good/Excellent	73	11.7	269	42.5	107	17.3	36	5.8
Total	212	24.1	511	57.1	283	32.3	113	12.9
ꭓ^2^ -statistic (df)	184.023 (1)		188.131 (1)		217.655 (1)		98.315 (1)	
*P*-Value	<0.001*		<0.001*		<0.001*		<0.001*	
Perceived mental health compared to before COVID-19								
Worse	155	39.5	315	78.9	194	50.0	81	20.9
Same/Better	57	11.7	196	39.4	89	18.2	32	6.6
Total	212	24.1	511	57.0	283	32.3	113	12.9
ꭓ^2^ -statistic (df)	91.954 (1)		140.989 (1)		100.095 (1)		39.497 (1)	
*P*-Value	<0.001*		<0.001*		<0.001*		<0.001*	
COVID-19 locus								
Distal (do not know any diagnosed person)	155	23.4	369	54.6	209	31.7	81	12.3
Proximal (related to a diagnosed person)	57	26.1	142	64.5	74	34.1	32	14.7
Total	212	24.1	511	57.0	283	32.3	113	12.9
ꭓ^2^ -statistic (df)	0.652 (1)		6.719 (1)		0.443 (1)		0.847 (1)	
*P*-Value	0.419		0.010*		0.506		0.357	
Employed								
No/Valid skip	95	25.6	219	58.4	133	36.0	48	13.1
Yes	117	23.1	288	55.7	149	29.4	65	12.8
Total	212	24.2	507	56.8	282	32.2	113	12.9
ꭓ^2^ -statistic (df)	0.721 (1)		0.643 (1)		4.251 (1)		0.016 (1)	
*P*-Value	0.396		0.423		0.039*		0.898	
Essential worker								
No/Valid skip/Don’t know	163	26.1	384	60.7	217	35.0	77	12.4
Yes	49	19.3	124	47.7	66	25.8	36	14.2
Total	212	24.1	508	56.9	283	32.3	113	12.9
ꭓ^2^ -statistic (df)	4.598 (1)		12.643 (1)		7.041 (1)		0.492 (1)	
*P*-Value	0.032*		<0.001*		0.008*		0.483	
Sense of belonging to the community								
Weak	146	34.8	304	70.7	187	44.3	78	18.8
Strong	64	14.0	202	43.8	94	20.8	33	7.3
Total	210	24.0	506	56.8	281	32.2	111	12.8
ꭓ^2^ -statistic (df)	51.842 (1)		65.507 (1)		55.019 (1)		25.709 (1)	
*P*-Value	<0.001*		<0.001*		<0.001*		<0.001*	
Accessed emotional and mental health resources								
No	120	18.4	322	48.5	162	24.9	43	6.6
Yes	92	41.4	185	81.9	120	53.8	70	31.5
Total	212	24.2	507	57.0	282	32.3	113	13.0
ꭓ^2^ -statistic (df)	48.009 (1)		76.564 (1)		63.365 (1)		91.080 (1)	
*P*-Value	<0.001*		<0.001*		<0.001*		<0.001*	

Source: Survey on COVID-19 and Mental Health (2020/2021).

^a^ = One of the cells in the crosstabulation between indigenous identity and post-traumatic stress disorder (PTSD) did not meet the required cell count by Statistics Canada. * = chi-statistics is significant at *p* < 0.05 (two tailed test). Bivariate estimates are based on available case analysis; missing cases were not accounted for.

### Multivariate Analysis

The two-way ANOVA results (footnote of Table 4) showed a significant gender difference in all of the mental health outcomes, with women having a higher prevalence of symptoms than men (*p* < 0.001). However, Table 4 (ANCOVA) showed that only the gender differences in anxiety (*F* = 6.700, *p* = 0.010, Adjusted *R*^*2*^ = 0.549) and psychological distress (*F* = 6.502, *p* = 0.011, Adjusted *R*^*2*^ = 0.577) remain after controlling for the twelve covariates; women had higher symptoms in both cases. In terms of areas of residence, rural dwellers had a significantly lower prevalence of psychological distress (*F* = 8.603, *p* = 0.003) and depression (*F* = 11.583, *p* = 0.001) than their urban counterparts. The gender*area of residence interaction effect was not significant for any of the outcomes. Figure 1 shows the estimated marginal means of the gender and area of residence interactions.

**Table 4.** Two-Way ANCOVA for Gender and Area of Residence Effects on Mental Health Outcomes

Variable	Anxiety		Distress		Depression		PTSD	
	F	*p*-value	F	*p*-value	F	*p*-value	F	*p*-value
Corrected model	39.600	<0.001*	62.999	<0.001*	48.833	<.001*	17.955	<0.001*
Intercept	49.228	<0.001*	87.778	<0.001*	69.553	<.001*	4.359	0.037*
Age	0.047	0.829	0.006	0.936	0.122	0.727	3.158	0.076
Household size	1.373	0.242	0.175	0.676	2.150	0.143	0.078	0.780
Total household income	0.000	0.994	0.941	0.332	2.214	0.137	4.436	0.036*
Immigrant status	2.347	0.126	0.292	0.589	0.006	0.939	2.180	0.140
Visible minority	0.899	0.343	0.058	0.810	0.112	0.738	4.298	0.039*
Assessed mental health services	16.122	<0.001*	55.253	<0.001*	27.662	<0.001*	56.100	<0.001*
Perceived mental health	44.877	<0.001*	52.520	<0.001*	36.956	<0.001*	20.284	<0.001*
Mental health compared to pre-COVID-19	37.751	<0.001*	44.941	<0.001*	19.897	<0.001*	2.691	0.101
COVID19 locus	0.300	0.584	0.030	0.862	0.433	0.511	0.508	0.476
Essential worker	1.597	0.207	1.312	0.252	0.121	0.728	7.256	0.007*
Sense of belonging	9.806	0.002*	7.297	0.007*	16.571	<0.001*	4.413	0.036*
Satisfaction with life	66.778	<0.001*	130.467	<0.001*	122.761	<0.001*	10.322	0.001*
Gender	6.700	0.010*	6.502	0.011*	2.128	0.145	1.539	0.215
Area of residence	0.134	0.714	0.203	0.652	1.394	0.238	2.765	0.097
Gender *area of residence	2.234	0.136	0.103	0.749	1.145	0.285	3.546	0.060
**R Squared**	**0.471**		**0.586**		**0.525**		**0.289**	
**Adjusted R Squared**	**0.459**		**0.577**		**0.514**		**0.272**	

Source: Survey on COVID-19 and Mental Health (2020/2021).

Model Summaries for ANOVA:

**ANOVA for Anxiety**: Gender F (1, 874) = 31.727, *p* < 0.001, Pη^2^ = 0.035; Area of residence F (1, 874) = 3.433, *p* = 0.064, Pη^2^ = 0.004; Gender*Area of residence F (1, 874) = 0.371, *p* = 0.542, Pη^2^ < 0.001. R^2^ = .069 (Adjusted R^2^ = .066).

**ANOVA for Distress**: Gender F (1, 877) = 30.181, *p* < 0.001, Pη^2^ = 0.033; Area of residence F (1, 877) = 8.603, *p* = 0.003, Pη^2^ = 0.010; Gender*Area of residence F (1, 877) = .005, *p* = 0. 946, Pη^2^ < 0.001. R^2^ = .065 (Adjusted R^2^ = .061).

**ANOVA for Depression**: Gender F (1, 872) = 30.752, *p* < 0.001, Pη^2^ = 0.034; Area of residence F (1, 872) = 11.583, *p* = 0.001, Pη^2^ = 0.013; Gender*Area of residence F (1, 872) = .063, *p* = 0. 801, Pη^2^ < 0.001. R^2^ = .072 (Adjusted R^2^ = .068).

**ANOVA for PTSD**: Gender F (1, 869) = 23.180, *p* < 0.001, Pη^2^ = 0.026; Area of residence F (1, 869) = 0.14, *p* = 0.906, Pη^2^ <0.001; Gender*Area of residence F (1, 869) = .130, *p* = 0. 718, Pη^2^ < 0.001. R^2^ = .047 (Adjusted R^2^ = .044).

**Figure 1.**
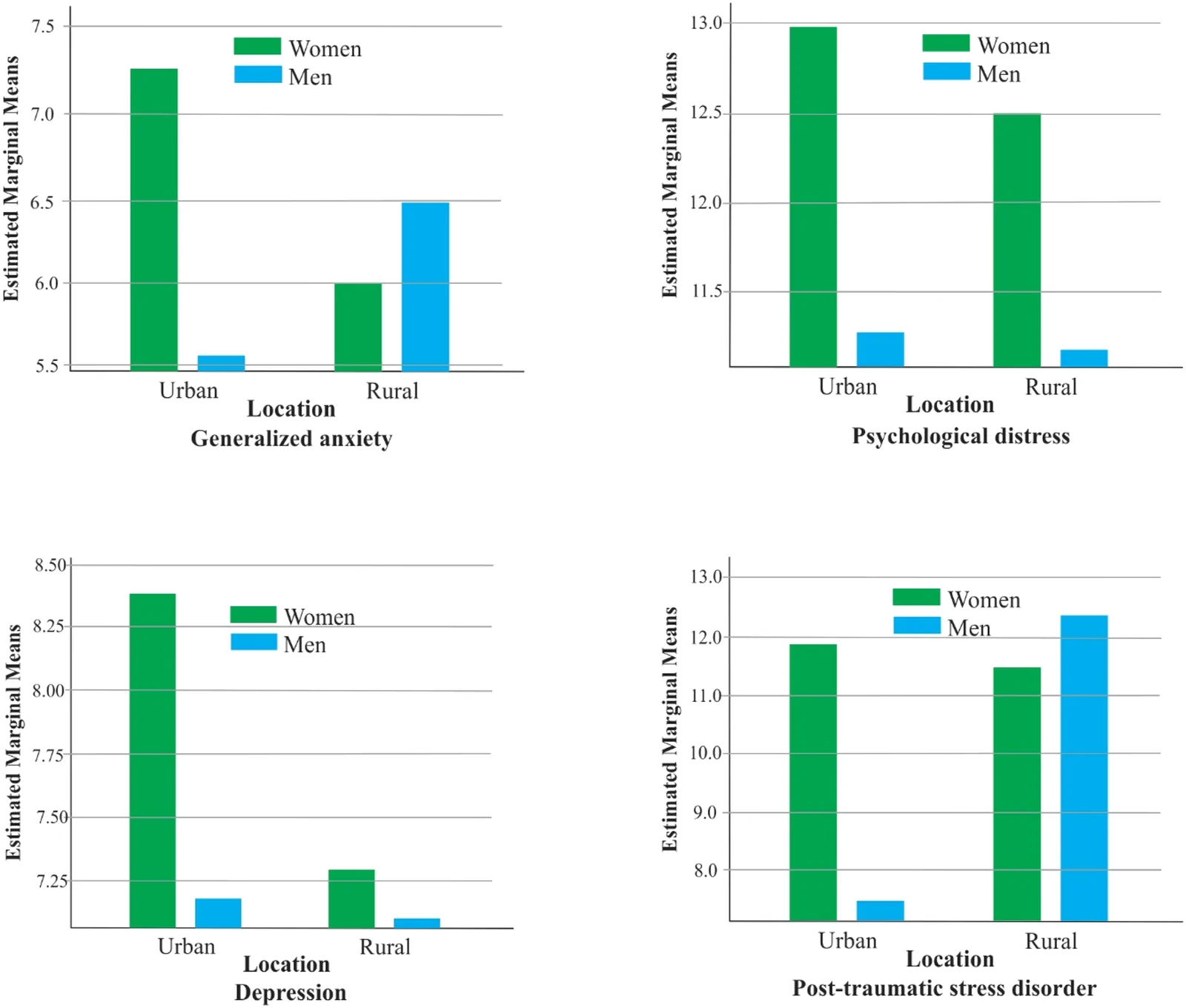
Two-way ANCOVA, estimated marginal means of the interaction effects of gender and area of residence on mental health outcomes. Source: Survey on COVID-19 and Mental Health (2020/2021)

Table 5 shows the multivariate linear regression models for sociodemographic correlates of each mental health outcome. Significant predictors for high anxiety were: being a woman (Standardized Regression Coefficients (*β*) = 0.13, *p* < 0.001), having a low community sense of belonging (*β* = −0.10, *p* = 0.003), accessing mental health care before COVID-19 (*β* = 0.12, *p* < 0.001), low self-perception of having a good mental health (*β* = −0.22, *p* < 0.001), poor mental health before COVID-19 (β = −0.19, *p* < 0.001), and a unit decrease in satisfaction with life in general (*β* = −0.30, *p* < 0.001). The model was robust (*F* [21, 665] = 27.495, *p* < 0.001) and accounted for 44.8% of the total variance.

**Table 5.** Multivariate Linear Regression for Sociodemographic Predictors of the Mental Health Outcomes

Parameter	Anxiety		Distress		Depression		PTSD	
	Beta (95% CI)	*p*-value	Beta (95% CI)	*p*-value	Beta (95% CI)	*p*-value	Beta (95% CI)	*p*-value
(Constant)	− (8.78, 17.8)	<0.001*	− (20.93, 33.63)	<0.001*	− (14.13, 24.11)	<0.001*	− (0.43, 30.69)	0.044*
Age of respondent (unit increase in years)	0.00 (−0.19, 0.18)	0.929	−0.01 (−0.31, 0.21)	0.717	−0.01 (−0.24, 0.17)	0.719	0.06 (−0.15, 1.09)	0.140
Gender of the respondent (Female)	0.13 (0.78, 2.09)	<0.001*	0.09 (0.69, 2.55)	<0.001*	0.08 (0.31, 1.77)	0.005*	0.11 (1.48, 5.94)	0.001*
Highest education (tertiary)	0.00 (−0.80, 0.74)	0.937	0.04 (−0.43, 1.75)	0.234	0.01 (−0.73, 0.99)	0.760	0.00 (−2.58, 2.62)	0.989
Area of residence (rural)	−0.01 (−0.96, 0.78)	0.838	−0.02 (−1.62, 0.83)	0.528	−0.04 (−1.66, 0.26)	0.154	0.06 (−0.35, 5.51)	0.084
Immigrant (permanent resident)	0.07 (−0.08, 2.13)	0.069	−0.01 (−1.75, 1.35)	0.797	0.04 (−0.57, 1.89)	0.293	−0.07 (−6.76, 0.65)	0.106
Non-permanent resident	−0.01 (−2.23, 1.43)	0.668	−0.02 (−3.54, 1.55)	0.444	−0.05 (−3.80, 0.21)	0.079	0.00 (−5.79, 6.41)	0.921
Indigenous identity (non-indigenous)	−0.01 (−1.55, 1.01)	0.681	−0.03 (−2.69, 0.94)	0.345	−0.03 (−2.11, 0.74)	0.347	−0.06 (−8.25, 0.42)	0.076
Visibility minority (non-visibility minority)	0.02 (−0.73, 1.20)	0.633	−0.01 (−1.63, 1.06)	0.679	−0.01 (−1.23, 0.91)	0.769	−0.08 (−6.04, 0.46)	0.093
COVID-19 locus (Proximal)	0.00 (−0.78, 0.73)	0.953	−0.01 (−1.20, 0.94)	0.815	0.02 (−0.60, 1.08)	0.574	0.02 (−1.83, 3.28)	0.579
Sense of belonging to the local community (Strong)	−0.10 (−1.75, −0.37)	0.003*	−0.08 (−2.32, −0.37)	0.007*	−0.12 (−2.32, −0.79)	<0.001*	−0.08 (−4.85, −0.18)	0.035*
Accessed emotions/mental health resources (Yes)	0.12 (0.77, 2.29)	<0.001*	0.19 (2.91, 5.05)	<0.001*	0.15 (1.46, 3.14)	<0.001*	0.24 (6.6, 11.73)	<0.001*
Perceived mental health (Better)	−0.22 (−3.53, −1.81)	<0.001*	−0.22 (−5.52, −3.08)	<0.001*	−0.20 (−3.76, −1.84)	<0.001*	−0.19 (−9.62, −3.71)	<0.001*
Mental health compared to before COVID-19 (Better)	−0.19 (−2.88, −1.45)	<0.001*	−0.19 (−4.50, −2.48)	<0.001*	−0.14 (−2.61, −1.02)	<0.001*	−0.07 (−4.57, 0.26)	0.080
Essential worker (No)	0.03 (−0.28, 1.11)	0.241	−0.03 (−1.52, 0.44)	0.280	0.01 (−0.64, 0.90)	0.743	0.09 (0.76, 5.47)	0.010*
Household size (unit increase per person)	0.02 (−0.16, 0.33)	0.483	0.01 (−0.28, 0.42)	0.707	0.03 (−0.15, 0.40)	0.356	0.02 (−0.61, 1.06)	0.592
Total household income (unit increase in $)	0.00 (0.00, 0.00)	0.994	−0.03 (0.00, 0.00)	0.366	−0.04 (0.00, 0.00)	0.212	−0.09 (0.00, 0.00)	0.019*
Satisfaction with life (unit increase)	−0.30 (−0.96, −0.59)	<0.001*	−0.37 (−1.78, −1.26)	<0.001*	−0.39 (−1.37, −0.96)	<0.001*	−0.13 (−1.65, −0.39)	0.001*
Atlantic region (Central)	0.05 (−0.23, 1.57)	0.146	0.04 (−0.52, 2.03)	0.246	0.02 (−0.63, 1.38)	0.464	−0.06 (−5.25, 0.87)	0.160
Prairie region (Central)	0.02 (−0.62, 1.08)	0.596	0.04 (−0.45, 1.96)	0.218	−0.02 (−1.17, 0.71)	0.626	−0.01 (−3.33, 2.42)	0.756
West coast region (Central)	0.05 (−0.24, 2.14)	0.117	0.02 (−1.04, 2.31)	0.458	0.03 (−0.62, 1.98)	0.305	0.00 (−3.81, 4.22)	0.919
North region (Central)	0.01 (−1.13, 1.32)	0.883	0.00 (−1.74, 1.75)	0.997	−0.03 (−2.16, 0.58)	0.258	0.04 (−1.83, 6.48)	0.273

Source: Survey on COVID-19 and Mental Health (2020/2021).

**Model summaries: Anxiety Model**: F (21, 665) = 27.495, *p* < 0.001. R = 0.682 (Adjusted R Squared = 0.448).

**Distress Model**: F (21, 664) = 45.139, *p* < 0.001. R = 0.767 (Adjusted R Squared = 0.575).

**Depression Model**: F (21, 660) = 35.552, *p* < 0.001. R = 0.729 (Adjusted R Squared = 0.516).

**PTSD Model**: F (21, 660) = 13.236, *p* < 0.001. R = 0.544 (Adjusted R Squared = 0.274).

Predictors for psychological distress and depression were the same factors as in anxiety; Table 5 showed the effect estimates. The model (*F* [21, 665] = 27.495, *p* < 0.001) accounted for 57.5% of the total variance in psychological distress. Similarly, the model summary for depression explained 51.6% of the total variance (*F* [21, 660] = 35.552, *p* < 0.001).

Table 5 showed that all the predictors of anxiety, psychological distress, and depression, including being an essential worker during the pandemic (*β* = 0.09, *p* = 0.010) and a unit decrease in household income (*β* = −0.90, *p* = 0.019), were significant predictors of PTSD. The model explained 27.4% of the total variance (*F* [21, 660] = 13.236, *p* < 0.001).

## Discussion

The study examined the prevalence and sociodemographic correlates of mental health symptoms among Canadian youth aged 18 to 24 years, focusing on the effect of gender and area of residence and their intersectionality on mental health outcomes. In the early stage of the pandemic, global research interest focused on mortality among older adults who were more vulnerable to COVID-19 (Findlay et al., 2020; Pérès et al., 2021). Beyond mortality, interests have shifted to the pandemic’s indirect effects, such as the rising prevalence of mental health disorders globally (Bourmistrova et al., 2022). The present study concurred with Gill et al. (2022), who reported that 60.1% of Canadian youths (18 to 24 years) meet the criteria for at least one of psychological distress, depression, anxiety, or PTSD. The result showed that 59.6% of the sample met at least one of these four symptoms. The pre-pandemic prevalence of mental health disorders among Canadian youths was of national concern (Statistics Canada, 2021a; Vaillancourt et al., 2021). Emerging data have shown a worsening trend following the pandemic (Bulloch et al., 2021; McGorry et al., 2025).

The discussion focused on the results of the multivariate-adjusted models. The ANCOVA models showed that females reported significantly higher anxiety and distress symptoms than their male counterparts. Virtually all the Canadian mental health studies of adolescents and youths supported the findings that mental health symptoms were more prevalent among women (Capaldi et al., 2022; Craig et al., 2022; Findlay et al., 2020; Salmon et al., 2022). It was expected that the impact of COVID-19 would be felt more by rural women (Mental Health Commission of Canada, 2021; Moffitt et al., 2022). Moffitt et al. (2022) suggested that rural women will be more vulnerable to the mental health impact of COVID-19 due to increased home violence, job loss, family chores, and childrearing responsibilities. Similarly, the Mental Health Commission of Canada (2021) posited that the unique influence of the social determinants of health in rural and remote communities makes the rural dwellers more vulnerable to the mental health impacts of COVID-19, as well as a lack of access to adequate and timely services and support. However, the estimated marginal means showed that rural residents had milder symptoms, especially for psychological distress and depression. This finding aligns with Capaldi et al. (2022), which showed that many people in rural areas reported better or stable mental health in 2020 and 2021 compared with urban dwellers. Whether facilitated by social supports, family, or a reduced prevalence of COVID-19 itself, there may have been a “rural advantage” for youths during this period. Another study among older adults found that people living in rural areas during COVID-19 experienced better social support, greater family presence, and fewer depressive and anxiety symptoms (Pérès et al., 2021). All the inferential statistics completed in the present study showed that gender was the primary sociodemographic correlate of mental health outcomes during the pandemic. Women had higher levels of anxiety, physiological distress, depression, and PTSD, especially in urban areas. The gendered finding aligned with other COVID-19 literature on Canadian youth reporting significantly higher prevalence of mental health disorders among women (Capaldi et al., 2022; Craig et al., 2022; Findlay et al., 2020; Salmon et al., 2022).

Aside from gender, the multivariate regression models showed other predictors of mental health symptoms, including being an essential worker, total household income, and psychosocial factors, such as a sense of belonging to the local community, mental health status before COVID-19, adverse change in mental health due to the pandemic, and satisfaction with life. Contrary to the assumption that essential workers would experience more mental health symptoms (Warren et al., 2021), essential workers and people from higher-income households reported less PTSD. The finding is congruent with other studies reporting that essential workers suffered less PTSD than the general population (Gill et al., 2022; Warren et al., 2021). Essential workers are in tune with their role expectations (Wang & Tang, 2020). While their workload and risk of infection increased, the perceived importance of their work, devotion to service, altruism, and heroism may have mitigated the adverse psychological effects (Wang & Tang, 2020). In line with other Canadian literature, this study found household income to be a significant predictor of young adults’ mental health (Salmon et al., 2022; Yu et al., 2021). Youths in low-income households may experience disproportionate traumatic health, social, and economic disturbances during the pandemic and have reduced access to mental health resources (Vaillancourt et al., 2021). Low-income households have been associated with an elevated risk for PTSD (Statistics Canada, 2024b), because economic disadvantage increases exposure to trauma, reduces access to protective and recovery resources, and contributes to chronic stress that heightens vulnerability to post-traumatic symptoms (Vaillancourt et al., 2021).

Psychosocial vulnerabilities are a determinant of mental health outcomes (Caron & Liu, 2010; Pérès et al., 2021). The present study has shown some sociodemographic and psychosocial factors relevant to post-pandemic surveillance. A weak community sense of belonging and decreased satisfaction with life (in general) negatively impacted all the mental health symptoms. Canadian young adults’ mental health was adversely affected by COVID-19-related social dynamics that impacted their interpersonal relationships, community cohesion, and ways of living (Gill et al., 2022; Hawke et al., 2020; Vaillancourt et al., 2021). Self-report of pre-existing mental health disorders, being enrolled in a mental health intervention before the pandemic, and perception of worsened mental health due to the pandemic were predictors of poor mental health. These findings agreed with Hawke et al. (2020), who reported a significant deterioration of mental health among Canadian youths in Ontario who had mental health illnesses before the COVID-19 pandemic. Similarly, Canadian studies have demonstrated that stronger community belonging is associated with higher life satisfaction and better mental health among youth, including those aged 15–29 (Foran, 2025) and 18–34 (Capaldi et al., 2022). However, our study specifically focused on youths aged 18–24.

Beyond the variables included in the present analysis, several pandemic-related contextual factors may further explain the elevated mental health symptoms observed among Canadian youth. Restrictions on outdoor physical activity and in-person social interaction, along with the resulting increase in digital engagement and screen time, have been consistently linked to higher levels of anxiety, depressive symptoms, sleep disturbances, and emotional dysregulation among adolescents and young adults (Chen et al., 2022; Santos et al., 2023). Prolonged reliance on online schooling and remote work has also been linked to cognitive overload, reduced academic structure, and heightened stress, especially among youth with limited access to supportive learning environments (Vaillancourt et al., 2021). In addition, a prior study on this cohort showed that social disruptions, including the death of a family member, friend, or colleague, feelings of loneliness, social isolation, emotional distress, and restrictions in personal relationships, negatively affected youth mental health during the pandemic (Onyeso et al., 2025). Although these factors were not directly measured in the present study, they likely served as salient contextual stressors and potential confounders during the pandemic, warranting explicit consideration in both clinical assessment and future research.

The exclusion of youth living on reserves and certain territories governed by distinct data-collection frameworks (The First Nations Information Governance Centre, 2019) may have resulted in conservative prevalence estimates or obscured some structural inequities affecting mental health outcomes. In our adjusted model, Indigenous youth showed higher levels of PTSD, while no statistically significant difference was observed in other mental health symptoms. Similarly, there was no significant association between youth mental health symptoms and immigration or minority status. This contrasts with broader Canadian evidence showing disproportionately higher rates of mental health disorders among Indigenous people, immigrants, and racialized adults, driven by structural inequities, historical and intergenerational trauma, systemic racism, and limited access to culturally safe services (Statistics Canada, 2024a). Our findings for immigrants and visible minority youth may differ from patterns typically observed in adults due to the characteristics of this cohort, namely its youth-specific composition (18–24 years), predominantly urban residence, and the rapid rise in mental health symptoms across the population during the pandemic, which may have reduced the ability to detect disparities at the population level. Nonetheless, inclusive surveillance systems and targeted research that centre the experiences of marginalized youth remain essential.

This study has some clinical and public health implications. The results support other studies that reported rising prevalence of mental health symptoms among Canadian youths (Bulloch et al., 2021; Findlay et al., 2020; Hawke et al., 2020; Li et al., 2023; Vaillancourt et al., 2021). From a clinical perspective, the observed associations point to intersecting risk pathways in which gender, socioeconomic disadvantage, pre-existing mental health conditions, and weakened community social cohesion increase vulnerability through cumulative stress exposure and reduced access to protective resources. At the same time, comparatively milder symptom profiles observed in some groups suggest the presence of resilience-promoting factors, including social support and economic stability, underscoring the importance of trauma-aware, socially informed clinical assessment and formulation that consider both vulnerability and resilience processes. Clinical psychotherapy and psychosocial interventions that strengthen coping capacity, social connectedness, and functional recovery may therefore play a key role in reducing symptom severity and supporting mental health among Canadian young adults.

From a public health perspective, surveillance efforts should prioritize urban-dwelling young women, individuals from lower-income households, and youth with prior mental health diagnoses or psychosocial disadvantage. The findings suggest a disproportionate concentration of mental health burden among young adults in urban settings, highlighting the need for targeted monitoring and intervention strategies. Community-based social interventions that enhance emotional support, foster positive relationships, and strengthen social cohesion should be integrated into public health responses. Consistent with the recommendations of Salmon et al. (2022), pandemic relief resources should be explicitly directed towards families and young adults at higher risk, such as families with young women and low-income households.

### Strengths and Limitations

The current study involved a multivariate analysis of a large national sample collected by a reliable federal government agency (Statistics Canada). Beyond the zero-order association between mental health symptoms and sociodemographic factors, the interaction effects were explored, while confounders were controlled within multivariate models.

However, the study has some limitations, such as using self-reported screening tools in lieu of clinical diagnosis of mental health disorders. Standardized self-reported screening tools assume a minimum level of health literacy; therefore, the quality of data obtained from youths may be influenced by variations in their literacy levels. Nonetheless, the instruments used in the study were screening tools whose reliability and validity are well-established, but the outcome should not be misconstrued as a clinical diagnosis. However, screening results are helpful for surveillance of population-based prevalence and justification for further clinical assessment and probable diagnosis of individuals. Although all Canadian provinces and territories were part of the survey, people living in federal reserves, institutions, and outside the capital cities in the territories were excluded from the survey due to a distinct data collection legislative framework and governance. Hence, the report was based on the analyzed samples and may not be generalizable to the entire Canadian population.

## Conclusion

There was a high prevalence of anxiety, psychological distress, depression, and PTSD symptoms among Canadian young adults during the COVID-19 pandemic. Mental health problems appeared to be higher among urban dwellers and females, irrespective of their locality. Essential workers reported less PTSD than non-essential workers and unemployed people during the pandemic. Being from a low-income household was a significant predictor of PTSD. Psychological determinants, such as low community sense of belonging and poor satisfaction with life, were associated with higher anxiety, psychological distress, depression, and PTSD scores. People with mental health problems before COVID-19 were significantly more affected than their counterparts. This study alerts the public health authorities to the impending post-pandemic rise in the prevalence of mental health disorders among Canadian young adults and vulnerable sub-populations. Interventions that enhance social connectedness, address socioeconomic stressors, and support adaptive coping may be particularly critical in mitigating long-term mental health consequences for youth exposed to large-scale public health crises. Screening and counseling services that are culturally safe and trauma-informed could be offered in schools and recreation centers, making them more accessible to youth.

## Data Availability

The datasets analyzed during the current study are accessible through the Statistics Canada microdata application portal https://www.statcan.gc.ca/en/microdata/data-centres/access.

## References

[bibr1-13591045261446034] Bourmistrova N. W. Solomon T. Braude P. Strawbridge R. Carter B. (2022). Long-term effects of COVID-19 on mental health: A systematic review. Journal of Affective Disorders, 299, 118–125. 10.1016/j.jad.2021.11.031 PMC8758130 34798148

[bibr2-13591045261446034] Bovin M. J. Marx B. P. Weathers F. W. Gallagher M. W. Rodriguez P. Schnurr P. P. Keane T. M. (2016). Psychometric properties of the PTSD checklist for diagnostic and statistical manual of mental disorders (PCL-5) in veterans. Psychological Assessment, 28(11), 1379–1391. 10.1037/pas0000254 26653052

[bibr3-13591045261446034] Bulloch A. Zulyniak S. Williams J. Bajgai J. Bhattarai A. Dores A. Lukmanji A. Pham T. Wiens K. Patten S. B. (2021). Poor mental health during the -19 pandemic: Effect modification by age. Canadian Journal of Psychiatry, 66(9), 827–829. 10.1177/0706743721994408 PMC8495305 33591213

[bibr4-13591045261446034] Capaldi C. A. Liu L. Ooi L. L. Roberts K. C. (2022). Self-rated mental health, community belonging, life satisfaction and perceived change in mental health among adults during the second and third waves of the COVID-19 pandemic in Canada. Health Promotion and Chronic Disease Prevention in Canada: Research, Policy and Practice, 42(5), 218–225. 10.24095/hpcdp.42.5.05 PMC9306317 35170929

[bibr5-13591045261446034] Caron J. Liu A. (2010). A descriptive study of the prevalence of psychological distress and mental disorders in the Canadian population: Comparison between low-income and non-low-income populations. Chronic Diseases in Canada, 30(3), 84–94. 20609292

[bibr6-13591045261446034] Chen Q. Dai W. Li G. Ma N. (2022). The impact of screen time changes on anxiety during the covid-19 pandemic: Sleep and physical activity as mediators. Sleep and Biological Rhythms, 20(4), 521–531. 10.1007/s41105-022-00398-1 PMC9202662 35729903

[bibr7-13591045261446034] Cornelius B. L. Groothoff J. W. van der Klink J. J. Brouwer S. (2013). The performance of the and GHQ-12 to screen for present state DSM-disorders among disability claimants. BMC Public Health, 13, 128. 10.1186/1471-2458-13-128 PMC3575398 23402478

[bibr8-13591045261446034] Craig S. G. Ames M. E. Bondi B. C. Pepler D. J. (2022). Canadian adolescents’ mental health and substance use during the COVID-19 pandemic: Associations with COVID-19 stressors. Canadian Journal of Behavioural Science, 55(1), 46–55. 10.1037/cbs0000305

[bibr9-13591045261446034] Findlay L. C. Arim R. Kohen D. (2020). Understanding the perceived mental health of Canadians during the COVID-19 pandemic. Health Reports, 31(4), 22–27. 10.25318/82-003-x202000400003-eng 32644764

[bibr46-13591045261446034] Foran H. (2025). Community and well-being: Exploring sense of belonging among youth. Statistics Canada. https://www150.statcan.gc.ca/n1/pub/75-006-x/2025003/article/00001-eng.htm.

[bibr10-13591045261446034] Gill P. K. Du C. Khan F. Karimi N. Sabharwal K. Agarwal M. (2022). The psychological effects of COVID-19 spread in young Canadian adults. International Journal of Social Psychiatry, 68(1), 216–222. 10.1177/0020764020988878 33611957

[bibr11-13591045261446034] Guerrero M. D. Barnes J. D. (2022). Profiles of mental health and their association with negative impacts and suicidal ideation during the COVID-19 pandemic: A Canadian perspective. Health Reports, 33(8), 19–30. 10.25318/82-003-x202200800002-eng 35984951

[bibr12-13591045261446034] Hawke L. D. Barbic S. P. Voineskos A. Szatmari P. Cleverley K. Hayes E. Relihan J. Daley M. Courtney D. Cheung A. Darnay K. Henderson J. L. (2020). Impacts of COVID-19 on youth mental health, substance use, and well-being: A rapid survey of clinical and community samples. Canadian Journal of Psychiatry, 65(10), 701–709. 10.1177/0706743720940562 PMC7502874 32662303

[bibr13-13591045261446034] Kessler R. C. Barker P. R. Colpe L. J. Epstein J. F. Gfroerer J. C. Hiripi E. Howes M. J. Normand S. L. Manderscheid R. W. Walters E. E. Zaslavsky A. M. (2003). Screening for serious mental illness in the general population. Archives of General Psychiatry, 60(2), 184–189. 10.1001/archpsyc.60.2.184 12578436

[bibr14-13591045261446034] King N. Pickett W. Rivera D. Byun J. Li M. Cunningham S. Duffy A. (2022). The impact of the COVID-19 pandemic on the mental health of first-year undergraduate students studying at a major Canadian university: A successive cohort study. The Canadian Journal of Psychiatry, 68(7), 499–509. 10.1177/07067437221094549 PMC9096012 35450455

[bibr15-13591045261446034] Kroenke K. Spitzer R. L. Williams J. B. (2001). The PHQ-9: Validity of a brief depression severity measure. Journal of General Internal Medicine, 16(9), 606–613. 10.1046/j.1525-1497.2001.016009606.x PMC1495268 11556941

[bibr16-13591045261446034] Li B. Ng K. Tong X. Zhou X. Ye J. Yu J. J. (2023). Physical activity and mental health in children and youth during COVID-19: A systematic review and meta-analysis. Child and Adolescent Psychiatry and Mental Health, 17(1), 92. 10.1186/s13034-023-00629-4 PMC10357657 37468975

[bibr17-13591045261446034] Ludwig-Walz H. Dannheim I. Pfadenhauer L. M. Fegert J. M. Bujard M. (2023). Anxiety increased among children and adolescents during pandemic-related school closures in Europe: A systematic review and meta-analysis. Child and Adolescent Psychiatry and Mental Health, 17(1), 74. 10.1186/s13034-023-00612-z PMC10286360 37344892

[bibr18-13591045261446034] Manea L. Gilbody S. McMillan D. (2012). Optimal cut-off score for diagnosing depression with the patient health questionnaire (PHQ-9). A Meta-Analysis, 184(3), E191–E196. 10.1503/cmaj.110829 PMC3281183 22184363

[bibr19-13591045261446034] McGorry P. Gunasiri H. Mei C. Rice S. Gao C. X. (2025). The youth mental health crisis: Analysis and solutions. Frontiers in Psychiatry, 15, 1517533. 10.3389/fpsyt.2024.1517533 PMC11790661 39906686

[bibr20-13591045261446034] Mental Health Commission of Canada . (2021). The impact of COVID-19 on rural and remote mental health and substance use. Retrieved from. https://file:///C:/Users/User/Dropbox/PC/Downloads/The-Impact-of-COVID-19-on-Rural-and-Remote-Mental-Health-and-Substance-Use.pdf.pdf.pdf

[bibr21-13591045261446034] Michaels C. Blake L. Lynn A. Greylord T. Benning S. (2022). Mental health and well-being ecological model. Center for Leadership Education in Maternal & Child Public Health University of Minnesota–Twin Cities. https://mch.umn.edu/resources/mhecomodel

[bibr22-13591045261446034] Moffitt P. Aujla W. Giesbrecht C. J. Grant I. Straatman A.-L. (2022). Intimate partner violence and COVID-19 in rural, remote, and Northern Canada: Relationship, vulnerability and risk. Journal of Family Violence, 37(5), 775–786. 10.1007/s10896-020-00212-x PMC7675398 33230368

[bibr23-13591045261446034] Newlove-Delgado T. Russell A. E. Mathews F. Cross L. Bryant E. Gudka R. Ukoumunne O. C. Ford T. J. (2023). Annual research review: The impact of COVID-19 on psychopathology in children and young people worldwide: Systematic review of studies with pre- and within-pandemic data. The Journal of Child Psychology and Psychiatry and Allied Disciplines, 64(4), 611–640. 10.1111/jcpp.13716 PMC10952503 36421049

[bibr24-13591045261446034] Onyeso O. K. Boco A. Islam M. K. Darku A. Hallstrom L. K. (2025). COVID-19 social and economic disruptions, mental health, and coping behaviours among young Canadians: A structural equation model. International Journal of Adolescence and Youth, 30(1), Article 2451674. 10.1080/02673843.2025.2451674

[bibr25-13591045261446034] Pérès K. Ouvrard C. Koleck M. Rascle N. Dartigues J. F. Bergua V. Amieva H. (2021). Living in rural area: A protective factor for a negative experience of the lockdown and the COVID-19 crisis in the oldest old population? International Journal of Geriatric Psychiatry, 36(12), 1950–1958. 10.1002/gps.5609 PMC8420248 34378244

[bibr26-13591045261446034] Premraj L. Kannapadi N. V. Briggs J. Seal S. M. Battaglini D. Fanning J. Suen J. Robba C. Fraser J. Cho S. M. (2022). Mid and long-term neurological and neuropsychiatric manifestations of post-COVID-19 syndrome: A meta-analysis. Journal of the Neurological Sciences, 434, Article 120162. 10.1016/j.jns.2022.120162 PMC8798975 35121209

[bibr27-13591045261446034] Roberts N. P. Kitchiner N. J. Lewis C. E. Downes A. J. Bisson J. I. (2021). Psychometric properties of the PTSD checklist for DSM-5 in a sample of trauma exposed mental health service users. European Journal of Psychotraumatology, 12(1), Article 1863578. 10.1080/20008198.2020.1863578 PMC8725778 34992744

[bibr28-13591045261446034] Royston P. Altman D. G. Sauerbrei W. (2006). Dichotomizing continuous predictors in multiple regression: A bad idea. Statistics in Medicine, 25(1), 127–141. 10.1002/sim.2331 16217841

[bibr29-13591045261446034] Salmon S. Taillieu T. L. Fortier J. Stewart-Tufescu A. Afifi T. O. (2022). Pandemic-related experiences, mental health symptoms, substance use, and relationship conflict among older adolescents and young adults from Manitoba, Canada. Psychiatry Research, 311, Article 114495. 10.1016/j.psychres.2022.114495 PMC8898590 35279437

[bibr30-13591045261446034] Santos R. M. S. Mendes C. G. Sen Bressani G. Y. de Alcantara Ventura S. de Almeida Nogueira Y. J. de Miranda D. M. Romano-Silva M. A. (2023). The associations between screen time and mental health in adolescents: A systematic review. BMC Psychology, 11(1), 127. 10.1186/s40359-023-01166-7 PMC10117262 37081557

[bibr31-13591045261446034] Schenker N. Raghunathan T. E. (2007). Combining information from multiple surveys to enhance estimation of measures of health. Statistics in Medicine, 26(8), 1802–1811. 10.1002/sim.2801 17278184

[bibr32-13591045261446034] Spitzer R. L. Kroenke K. Williams J. B. Löwe B. (2006). A brief measure for assessing generalized anxiety disorder: The -7. Archives of Internal Medicine, 166(10), 1092–1097. 10.1001/archinte.166.10.1092 16717171

[bibr33-13591045261446034] Statistics Canada. (2021a). Survey on COVID-19 and Mental Health, September to December 2020. Statistics Canada. https://www150.statcan.gc.ca/n1/daily-quotidien/210318/dq210318a-eng.htm

[bibr34-13591045261446034] Statistics Canada. (2021b). Survey on COVID-19 and Mental Health, February to May 2021. Statistics Canada. https://www150.statcan.gc.ca/n1/daily-quotidien/210927/dq210927a-eng.htm

[bibr35-13591045261446034] Statistics Canada . (2023). Survey on COVID-19 and Mental Health (SCMH). Statistics Canada. https://www23.statcan.gc.ca/imdb/p2SV.pl?Function=getSurvey&SDDS=5330.

[bibr36-13591045261446034] Statistics Canada . (2024a). Inequalities in mental health, well-being and wellness in Canada. Statistics Canada. https://health-infobase.canada.ca/mental-health/inequalities/

[bibr37-13591045261446034] Statistics Canada . (2024b). Posttraumatic stress disorder among adults in Canada. Statistics Canada. https://health-infobase.canada.ca/ptsd-survey/

[bibr38-13591045261446034] The First Nations Information Governance Centre . (2019). First Nations data sovereignty in Canada. Statistical Journal of the IAOS, 35(1), 47–69. 10.3233/sji-180478

[bibr39-13591045261446034] Thomas S. Wannell B. (2009). Combining cycles of the Canadian Community Health Survey. Health Reports, 20(1), 53–58. 19388369

[bibr40-13591045261446034] Vaillancourt T. Szatmari P. Georgiades K. Krygsman A. (2021). The impact of COVID-19 on the mental health of Canadian children and youth. FACETS, 6, 1628–1648. 10.1139/facets-2021-0078

[bibr41-13591045261446034] Wang G. Y. Tang S. F. (2020). Perceived psychosocial health and its sociodemographic correlates in times of the COVID-19 pandemic: A community-based online study in China. Infectious Diseases of Poverty, 9(1), 148. 10.1186/s40249-020-00770-8 PMC7586696 33106187

[bibr42-13591045261446034] Warren A. M. Bennett M. Danesh V. Waddimba A. Tovar M. Gottlieb R. L. Powers M. B. (2021). Early psychological health outcomes among United States healthcare professionals, essential workers, and the general population during the COVID-19 pandemic: The influence of occupational status. Psychiatry Research Communications, 1(2), Article 100005. 10.1016/j.psycom.2021.100005 PMC8559417 34977910

[bibr43-13591045261446034] World Health Organization . (2022). Health equity: Social determinant of health. World Health Organization. https://www.who.int/topics/health_equity/en/

[bibr44-13591045261446034] Xiong J. Lipsitz O. Nasri F. Lui L. M. W. Gill H. Phan L. Chen-Li D. Iacobucci M. Ho R. Majeed A. McIntyre R. S. (2020). Impact of COVID-19 pandemic on mental health in the general population: A systematic review. Journal of Affective Disorders, 277, 55–64. 10.1016/j.jad.2020.08.001 PMC7413844 32799105

[bibr45-13591045261446034] Yu L. Lecompte M. Zhang W. Wang P. Yang L. (2021). Sociodemographic and COVID-related predictors for mental health condition of mainland Chinese in Canada amidst the pandemic. International Journal of Environmental Research and Public Health, 19(1), 171. 10.3390/ijerph19010171 PMC8750305 35010431

